# Bladder cancer index: cross-cultural adaptation into Spanish and psychometric evaluation

**DOI:** 10.1186/1477-7525-12-20

**Published:** 2014-02-15

**Authors:** Stefanie Schmidt, Ricard Riel, Albert Frances, José Antonio Lorente Garin, Xavier Bonfill, María José Martinez-Zapata, Maria Morales Suarez-Varela, Javier dela Cruz, José Ignacio Emparanza, María-José Sánchez, Javier Zamora, Juan Manuel Ramos Goñi, Jordi Alonso, Montse Ferrer

**Affiliations:** 1 Health Services Research Group, IMIM (Hospital del Mar Medical Research Institute), Doctor Aiguader 88, 08003 Barcelona, Spain; 2 Department of Experimental and Health Sciences, Universidad Pompeu Fabra (UPF), Barcelona, Spain; 3 CIBER Epidemiología y Salud Pública (CIBERESP), Madrid, Spain; 4 Center of Primary Health Care El Clot, Barcelona, Spain; 5 Department of Urology, Hospital del Mar, Barcelona, Spain; 6 Iberoamerican Cochrane Centre, Biomedical Research Institute Sant Pau (IIB Sant Pau), Barcelona, Spain; 7 Universitat Autònoma de Barcelona (UAB), Barcelona, Spain; 8 Unit of Public Health and Environmental Care, Department of Preventive Medicine, University of Valencia, Valencia, Spain; 9 Center for Public Health Research (CSISP), Valencia, Spain; 10 Hospital 12 de Octubre, Madrid, Spain; 11 Clinical Epidemiology Unit, Hospital Universitario Donostia, BioDonostia, San Sebastian, Spain; 12 Escuela Andaluza de Salud Pública, Granada, Spain; 13 Instituto de Investigación Biosanitaria de Granada (Granada.bs), Granada, Spain; 14 Clinical Biostatistics Unit, Hospital Ramón y Cajal (IRYCIS), Madrid, Spain; 15 HTA Unit of the Canary Islands Health Service (SESCS), S/C de Tenerife, Spain; 16 Health Services Research on Chronic Patients Network (REDISSEC), Bilbao, Spain

**Keywords:** Urinary bladder neoplasms, Quality of life, Patient outcomes, Validation studies, Psychometrics

## Abstract

**Background:**

The Bladder Cancer Index (BCI) is so far the only instrument applicable across all bladder cancer patients, independent of tumor infiltration or treatment applied. We developed a Spanish version of the BCI, and assessed its acceptability and metric properties.

**Methods:**

For the adaptation into Spanish we used the forward and back-translation method, expert panels, and cognitive debriefing patient interviews. For the assessment of metric properties we used data from 197 bladder cancer patients from a multi-center prospective study. The Spanish BCI and the SF-36 Health Survey were self-administered before and 12 months after treatment. Reliability was estimated by Cronbach’s alpha. Construct validity was assessed through the multi-trait multi-method matrix. The magnitude of change was quantified by effect sizes to assess responsiveness.

**Results:**

Reliability coefficients ranged 0.75-0.97. The validity analysis confirmed moderate associations between the BCI function and bother subscales for urinary (r = 0.61) and bowel (r = 0.53) domains; conceptual independence among all BCI domains (r ≤ 0.3); and low correlation coefficients with the SF-36 scores, ranging 0.14-0.48. Among patients reporting global improvement at follow-up, pre-post treatment changes were statistically significant for the urinary domain and urinary bother subscale, with effect sizes of 0.38 and 0.53.

**Conclusions:**

The Spanish BCI is well accepted, reliable, valid, responsive, and similar in performance compared to the original instrument. These findings support its use, both in Spanish and international studies, as a valuable and comprehensive tool for assessing quality of life across a wide range of bladder cancer patients.

## Background

Bladder cancer is one of the most complex neoplasms in urologic oncology. In men it is the fourth leading cancer location in the European Union and the United States [1,2]. Estimated incidence rates for men are lower in Europe than in the United States (29.1 vs 37.6 per 100,000); however, the rate for Spanish males is among the highest in the European Union (39.0 per 100,000). Spanish men present about eight times higher incidence rates than Spanish women [1].

Health-related quality of life (HRQL) is an important outcome for evaluating the impact of disease and for monitoring treatment benefits and side effects. Since bladder cancer survivors have usually undergone several treatments, measuring HRQL with valid instruments is of value for clinicians and patients when making informed decisions based on patients’ experiences [3]. Although HRQL assessment is an essential endpoint in clinical trials [4] and comparative effectiveness research [5], it is still infrequently used in bladder cancer studies [6].

Almost all of the specific HRQL questionnaires available for bladder cancer were either developed by the European Organisation for Research and Treatment of Cancer (EORTC) or the American organization for Functional Assessment of Cancer Therapy (FACT). Both recommend measuring HRQL with their general wellbeing core questionnaire for oncologic patients plus a specific module. The EORTC-QLQ-BLS24 and the FACT-Bladder modules are applicable for patients with superficial bladder cancer; while the EORTC-QLQ-BLM30 and the FACT Vanderbilt Cystectomy Index are for patients at muscle invasive stage [7-9]. These modules were designed for specific grades of tumor infiltration and types of treatment (mainly transurethral resection or cystectomy), which leads to certain difficulties when dealing with mixed patient characteristics in clinical practice or with comparative research on effectiveness.

The Bladder Cancer Index (BCI) [10,11] was developed in the United States (2007) to overcome this limitation, as it contains neutral questions regarding native or neo-bladder, urinary diversion method, and gender. It is therefore comprehensive across a wide range of bladder cancer patients, independent of tumor infiltration and treatment applied. It has shown to be a robust multidimensional HRQL measure. The BCI development process included a literature review, an expert panel study, and input from bladder cancer survivors who reviewed the content before pilot testing. The BCI demonstrated high internal consistency and test-retest reliability, interscale independence among domains [11] and different HRQL profiles among treatments [10].

HRQL is a standard outcome in clinical trials [4], with increasing need for available measures in different languages to perform international multi-center studies. Therefore, the aim was to linguistically and culturally adapt the BCI for its use in Spain, and to test the acceptability, reliability, validity, and responsiveness of this adapted version.

## Methods

### The Bladder Cancer Index (BCI)

The BCI consists of 36 items, with 4- or 5-point Likert response scales, covering 3 primary domains: urinary (14 items), bowel (10 items), and sexual (12 items). For each domain a summary score and two subscale scores (function and bother) are constructed. The function items focus on the frequency of the disease symptoms, with answer scales such as: “Never, rarely, about half the time, usually, or always”. The items of bother reflect the individual perception of these symptoms, usually categorized as: “No problem, very small, small, moderate, or big problem”. Following the algorithm developed by the authors of the original instrument [11], scores are calculated by transforming item responses into a 0 to 100 scale and calculating the mean of the standardized items. Higher scores indicate better health status. To calculate a score, a minimum of 80% completed items is required.

### Linguistic and cultural adaptation

Standard methods were used to translate and culturally adapt the instrument [12]. The Spanish translation of the BCI was carried out independently by two professional linguists, both native Spanish speakers, with a high level of fluency in English. The focus of these forward translations was achieving a conceptual, rather than literal, equivalence. Afterwards, an interdisciplinary group of researchers (two experts in quality of life assessment, an urologist, and an oncologic nurse) reviewed the two BCI translations and reached a consensus version.

Cognitive debriefing interviews were held to explore the understandability of this preliminary version, and to identify discrepancies with the original BCI. Individualized interviews were carried out with 11 patients (9 of which had non-muscle invasive bladder cancer and 2 had muscle-invasive disease) who were aged 54-82 years old. This technique allowed assessing what the patient understood in the adapted version. Only minor changes were included as a result of patients’ feedback because they found the Spanish BCI version to be understandable and adequate. The resulting modification was the omission of brand names, as most Spanish patients are not aware of the name of their specific urinary diversion.

As a last step, this pre-final version was translated back into English by a native American-English speaker. The original and back-translated versions were compared and sent to the author of the original BCI for evaluation. Since no major discrepancies were found, no changes were introduced in the final Spanish version.

### Study of metric properties

The psychometric properties of the questionnaire were tested in a subsample of bladder cancer patients from a multi-center prospective study. This study was conducted from October 2010 to September 2011 and focused on the clinical care process and health outcomes of patients with urologic tumors. Briefly, patients were consecutively enrolled from the urologic departments of 7 hospitals in 5 Spanish autonomous regions. The inclusion criteria were 1) having an anatomopathological confirmation of bladder cancer during the study period, 2) being diagnosed and treated in one of the study hospitals, and 3) agreeing to participate in the study and to sign an informed consent form. The study was approved by the corresponding ethic committees.

Clinical data were retrieved from medical records, and HRQL data were collected before and 12 months after treatment. Patients self-completed the SF-36 and the BCI during their outpatient visits. The short-form health questionnaire SF-36 (version 2) [13] is a 36-item generic HRQL questionnaire covering eight dimensions, which can be summarized into a physical and a mental component summary score (PCS and MCS, respectively). Summary scores are standardized to have a mean of 50 and a standard deviation of 10 in the U.S. general population [13]. Scores above or below 50 indicate better or worse health status compared to the general population. The post-treatment interview additionally included a question on global health change: “How would you rate your current bothers related with your bladder tumor compared to those before treatment (1 year ago)? You feel better; You feel the same; You feel worse”. Those patients who reported complete HRQL data at baseline and 12 months after treatment, as well as the question on global change, were selected to compose the BCI validation subsample. Its sample size (n = 197) gave a statistical power of 0.8 to detect small differences of five points on the urinary summary score between pre- and post-treatment, using a two-sided paired t-test with a type I error of 5%.

### Statistical analysis

Mean, standard deviations, score range, and percentage of patients with the worst possible (floor effect) and best possible theoretical scores (ceiling effect) were calculated in order to examine the score distribution. Cronbach’s alpha coefficient was calculated to assess reliability based on internal consistency [14]. To provide the most similar comparison possible with the original BCI study, all the analyses were conducted with the 12 month post-treatment data, except for the responsiveness evaluation, where pre- and post-treatment data were used.

To assess construct validity, interscale correlations (Pearson coefficients) between the BCI domains and subscales and with the SF-36 scores (multi-trait multi method matrix) were calculated. Pre-specified hypothesis were that: a) Function and bother subscales within each individual BCI domain present moderate correlation, as bother subscales quantify the symptoms’ impact, measured by the function subscales; b) In contrast, correlations among different BCI domains are low since urinary, sexual, and bowel domains measure different HRQL components; and c) Correlations between BCI and SF-36 scores are moderate to low, due to differences between generic and disease-specific instruments. Correlations of <0.45, 0.45-0.70, and >0.70 were considered as low, moderate, and high, respectively [15].

To evaluate responsiveness, pre- and post-treatment mean scores were compared using a paired t-test among patients reporting improvement in the global health change question. To quantify the magnitude of change, effect sizes were calculated as the mean score differences divided by the standard deviation of pre-treatment scores. Effect sizes of 0.2, 0.5 and 0.8 were defined as small, moderate and large, respectively [16]. Analyses were carried out with SPSS statistics software, version 12 (SPSS, Chicago, IL, USA).

## Results

Table 1 shows the clinical and demographic characteristics of the 197 patients with bladder cancer who composed the BCI validation subsample. Patients were mainly men (86.8%) with a mean age of 69 years. Transitional cell carcinoma was the most prevalent (70%) and 84% of patients were diagnosed at non-muscle invasive stages (Ta, Tis, or T1). Transurethral resection (TUR) was the primary treatment applied (96.3%), in some cases combined with either Bacillus Calmette–Guérin (17.4%) or intravesical chemotherapy (12.6%). During the follow-up, three patients developed metastasis (two to the lung and one lymphatic) and 36 patients presented cancer recurrence or progression (18.3%). At the end of the study, 23 of these patients were in complete remission.

**Table 1:** Demographic and clinical characteristics of bladder cancer patients

	**N (%)**
**Total patients**	197
**Age**	
Mean (*standard deviation*)	69.3 (*11*)
**Sex**	
Male	171 (86.8)
Female	26 (13.2)
**Tumor histology**	
Adenocarcinoma	17 (10.2)
Transitional cell carcinoma	116 (69.9)
Squamous-cell carcinoma	3 (1.8)
Others	30 (18.1)
* Missing*	*31 (15.7)*
**Disease stage**	
Tx	5 (2.5)
Ta	58 (29.4)
Tis	5 (2.5)
T1	102 (51.8)
T2a	16 (8.1)
T2b	6 (3.0)
T3	3 (1.5)
T4	2 (1.0)
* Missing*	*11 (5.6)*
**Medical treatment**	
Transurethral resection	183 (96.3)
Radical cystectomy	6 (3.2)
Bacillus Calmette–Guérin	33 (17.4)
Chemotherapy	24 (12.6)
Radiotherapy	3 (1.6)
* Missing*	*7 (3.6)*
**Education**	
Incomplete studies	62 (31.5)
Primary or secondary studies	103 (52.6)
Superior studies	31 (15.9)

The percentage of patients with any missing item in urinary, bowel or sexual domains was 15.7%, 7.6% and 17.8%, respectively (Table 2). The proportion of insufficient information to calculate the score (missing items > 20%) was the highest for the sexual (10.7-12.7%) and the lowest for the bowel domain (2.5-4.1%). No floor effect was found except for the sexual function domain (33%). Ceiling effects were observed in all domains, being the highest in the urinary function subscale (71.4%), and the lowest for the sexual summary (2.9%). All Cronbach’s alpha values were high, ranging 0.75-0.97.

**Table 2:** BCI scores distribution and internal consistency

**BCI domains**	**N° items**	**Mean (SD)**	**Missing items**	**Missing score**	**Observed range**	**Floor effect**	**Ceiling effect**	**Cronbach’s alpha**
**Urinary**	14	88.8 (19.3)	15.7	8.6	0 – 100	0.6	50.0	0.92
Function	6	88.2 (24.4)	7.6	7.6	0 – 100	2.7	71.4	0.91
Bother	8	89.2 (20.0)	13.7	10.2	0 – 100	0.6	58.2	0.90
**Bowel**	10	90.1 (14.7)	7.6	2.5	13.9 – 100	0	34.4	0.84
Function	4	92.4 (15.1)	4.1	4.1	6.3 – 100	0	57.7	0.75
Bother	6	89.0 (17.2)	6.1	3.0	20.0 – 100	0	51.3	0.78
**Sexual**	12	51.5 (24.0)	17.8	12.7	6.8 – 100	0	2.9	0.88
Function	7	31.2 (32.1)	15.2	10.7	0 – 100	33.0	4.0	0.97
Bother	5	79.3 (29.8)	15.2	12.7	0 – 100	0.6	58.1	0.86

Missing items: percentage of patients with any missing items; Missing score: percentage of patients with any missing score; Floor effect: percentage of patients with worst possible score (0); Ceiling effect: percentage of patients with best possible score (100).

Table 3 shows the multi-trait multi-method matrix of correlations with the BCI and with the SF-36 scores. As previously hypothesized, the strength of the association between symptom severity (function subscale) and its impact (bother subscale) was moderate for each specific BCI domain, with Pearson coefficients of 0.61 and 0.53 marked in bold. Only for the sexual domain we observed a low correlation (r = 0.15). BCI domain subscales presented low correlations (<0.30) with the other BCI domains. Finally, most of the correlation coefficients between BCI and SF-36 scores were lower than 0.40.

**Table 3:** Correlations among BCI subscales and SF-36 summary component scores

		**Bladder Cancer Index (BCI)**					
		**Urinary**		**Bowel**		**Sexual**	
**B C I**		Function	Bother	Function	Bother	Function	Bother
	**Urinary**						
	Function	1					
	Bother	**0.61**	1				
	**Bowel**						
	Function	0.09	0.26	1			
	Bother	0.11	0.32	**0.53**	1		
	**Sexual**						
	Function	0.27	0.28	0.14	0.26	1	
	Bother	0.05	0.12	0.10	0.11	**0.15**	1
**S F – 3 6**	**SF-36**						
	PSC	0.28	0.44	0.23	0.34	0.28	0.14
	MSC	0.24	0.48	0.27	0.36	0.19	0.16

Correlations (Pearson coefficients) previously hypothesized as moderate are marked in bold.

**Figure 1:**
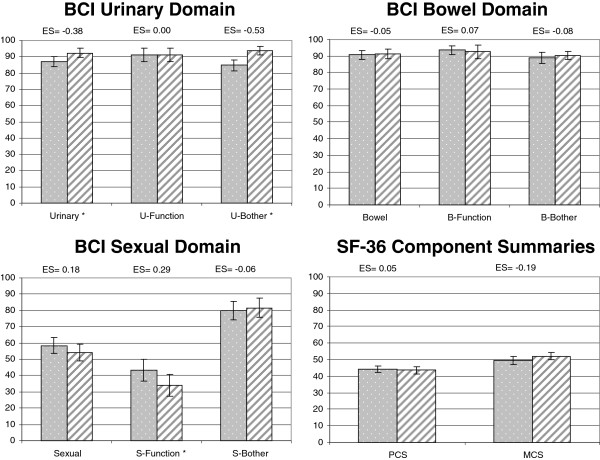
**Responsiveness to health change in patients who reported improvement 12 months after treatment (n = 110).** Footnote: Bars with points reflect the pre-treatment scores; bars with stripes the post-treatment scores. Confidence interval 95%. *indicate statistical significance between pre- and post-treatment scores with p < 0.05. ES: effect size.

Responsiveness was evaluated in the group of 110 patients who reported improvement 12 months after treatment (Figure 1). The median follow-up time between pre and post-treatment evaluation was 424 days. Change between pre- and post-treatment indicated a statistically significant improvement in the BCI urinary summary (effect size =0.38, p =0.003) and urinary bother subscale (effect size =0.53, p <0.001). No statistically significant changes were observed on bowel scores. Sexual function showed statistically significant worsening of small magnitude (effect size =0.29, p =0.009).

## Discussion

We used a standard cross-cultural adaptation process to develop the Spanish BCI version, which demonstrated a good patient acceptability, high reliability, good construct validity, and sensitivity to change over time. The results are consistent with those obtained for the original BCI and suggest that the Spanish version is conceptually and metrically equivalent.

Regarding acceptability, the relatively high percentage of patients with any missing items (27%) may indicate some problems. However, the number of missing items per patient was low, with a mean of 2.2 (standard deviation = 5), and the percentage of missing per item ranges from 0.5% to 14% of patients. The fact that missing values were not concentrated in specific items or domains supports the idea that the BCI did not include any unsuitable or irrelevant item, and that it was well accepted by Spanish patients. In terms of reliability, as Cronbach’s alpha coefficients were above the standard of 0.7 [14], all summary and subscale scores can be used for comparing groups of patients. Urinary scores achieved the more demanding standard of 0.9 for individual comparisons (individual change over time, or differences among individuals). These results are very similar to the reliability coefficients reported for the original version (Cronbach’s alpha 0.77-0.94).

The high ceiling effect observed on urinary and bowel domains, especially for the function subscale, is congruent with the clinical characteristics of our patient sample. The maximum score means good function and no bother, which is the case when dealing with patients diagnosed at a superficial disease stage, as in more than 80% of our sample. The ceiling effect for the sexual domain was specially marked on the bother subscale, where almost 60% of patients reported no sexual bother. Ceiling effects reported by the study of the original instrument were also mainly on the function subscale for urinary domain and on bother subscale for sexual domain.

The association between sexual function and bother subscales deserves a comment because it was unexpectedly low compared with the original study. A previous study assessing country differences in localized prostate cancer [17] showed that a higher percentage of patients in Spain tend to report low sexual functioning than in the USA. Our patients also presented low sexual functioning and did not perceive this dysfunction as a bother. Although cross-cultural differences should not be discarded, it may be due to the fact that sexual problems could have appeared some time ago and become accepted as a “normal” consequence of ageing, not relating them to bladder cancer or its treatment.

Unlike prior FACT Bladder or FACT Vanderbilt Cystectomy Index specific modules, which only provide an overall score [8], BCI allows separate scores for the three distinct domains facilitating a more detailed HRQL profile of bladder cancer disease impact. The conceptual independence among urinary, bowel, and sexual domains was supported by the low interscale correlations (ranging from 0.05 to 0.32), which were very similar to those reported by the original version (range 0.17-0.39). The low correlations obtained with the SF-36 suggest that BCI captures additional information which is not covered by generic instruments.

The moderate urinary changes observed between pre- and post-treatment evaluations of patients perceiving improvement after treatment demonstrate the BCI’s responsiveness over time. Furthermore, the high percentage of patients diagnosed at initial stages and treated with minimal invasive techniques (i.e. endoscopic removal of cancerous tissue) explains the small sexual worsening and the bowel stability observed. These results are consistent with the original BCI cross-sectional study comparing groups with different surgical approaches [11].

Some study limitations deserve further comment. First, our study design differed substantially from the design of the original BCI study. Gilbert et al [9,10] obtained the HRQL assessment of patients at 1 to10 years after diagnosis, while our HRQL evaluation was performed 1 year after. Second, cancer stage homogeneity of our sample (84% with non-muscle invasive disease) limits the generalizability of results to patients with advanced disease, and did not allow a comparison among different therapeutic groups. However, results from the original USA study, with a sample composed by 40% of patients at muscle-invasive disease stages and 70% with high grade tumors, support the suitability of BCI across the wide spectrum of this disease. Third, because our study mainly included men, generalizing our results to women with bladder cancer is uncertain. Finally, our study design did not allow test-retest analysis to assess the questionnaire’s repeatability, but the BCI’s high internal consistency supports adequate reliability.

## Conclusions

Researchers and clinicians now have at their disposal a bladder cancer-specific HRQL instrument for use in Spanish patients that is applicable across the wide spectrum of this disease. Our results suggest the multi-dimensionality of the Spanish BCI version, and provide considerable evidence about its appropriate metric properties, including responsiveness to health changes over time even in patients treated with non-invasive techniques. Comparison with the original U.S. version shows that it is similar in reliability and validity, suggesting that the cross-cultural adaptation method followed has yielded an equivalent Spanish version. Moreover, proofs supporting the BCI as a valuable tool for assessing HRQL in patients within the whole bladder cancer spectrum are strengthened by the demonstration of its appropriateness in a different language and culture [18] and reinforces its usefulness for international studies.

### Ethical committee approval

The study was approved by all the research ethic committees of the participating centers (Fundació Puigvert-Hospital de la Santa Creu i Sant Pau, Hospital del Mar, Hospital Universitario 12 de Octubre, Hospital Universitario Ramón y Cajal, Hospital Universitario Donostia, Hospital General Universitario de Valencia, and Hospital Universitario Virgen de las Nieves).

## Abbreviations

BCI: Bladder cancer index; EORTC: European Organisation for Research and Treatment of Cancer; EORTC-QLQ-BLM30: EORTC quality of life muscle-invasive bladder cancer; EORTC-QLQ-BLS24: EORTC quality of life superficial bladder cancer; FACT: American organization for Functional Assessment of Cancer Therapy; HRQL: Health-related quality of life; SF-36: Short-form Health Survey 36.

## Competing interests

The authors declare that they have no competing interests.

## Authors’ contributions

All authors have actively participated in the study and have made a substantial contribution to (1) either conception and design, or acquisition of data, or analysis and interpretation of data; as well as (2) the drafting of the article or its critical revision for important intellectual content; and (3) to the final approval of the version to be published. Each author believes that the manuscript represents honest work.

## EMPARO-CU Study Group

**Barcelona:** Virginia Becerra, Stefanie Schmidt, Yolanda Pardo, Montserrat Ferrer Fores, Olatz Garin **(IMIM Hospital del Mar Medical Research Institute);** Albert Frances **(Hospital del Mar);** Carola Orrego Villagran, Rosa Suñol **(Instituo U. Avedis Donabedian);** Dimelza Osorio, Xavier Bonfill Cosp, Esther Canovas, Gemma Sancho Pardo. Ignasi Bolívar, Jordi Bachs, José Pablo Maroto, Mª Jesús Quintana, Mª José Martínez Zapata, Cristina Martin Lorente **(Hospital de la Santa Creu i Sant Pau);** Ferran Algaba, Palou Redorta, Salvador Esquena **(Fundació Puigvert);** Robin Vernooij **(Centro Cochrane Iberoamericano). Bilbao:** Amaia Martínez, José Ignacio Pijoan Zubizarreta **(Hospital de Cruces)**; Lorea Martínez **(CAIBER Cruces). Canarias:** David Manuel Castro Diaz **(SCS HUC)**; Juan Manuel Ramos Goñi **(SESCS)**; Julio López Bastida **(Servicio Canario de la Salud). Granada:** Armando Suárez Pacheco, Cesar García López, Jose Manuel Cozar Olmo **(Hosp. U. Virgen de las Nieves)**; Carmen Martínez, Daysy Chang Chan, Mª Jose Sanchez Perez **(Escuela Andaluza de Salud Pública). Madrid:** Ana Isabel Díaz Moratinos, Angel Montero Luis, Asunción Hervás, Carmen Vallejo Ocaña, Costantino Varona, Javier Burgos, Javier Zamora, Jose Alfredo Polo Rubio, Luis López-Fando Lavalle, Miguel Angel Jimenez Cidre, Alfonso Muriel Garcia, Nieves Plana Farras, Rosa Morera Lopez, Sonsoles Sancho Garcia, Victor Abraira, Victoria Gomez Dos Santos **(Hosp. Ramón y Cajal)**; Agustín Gómez de la Cámara, Javier de la Cruz, Juan Passas Martinez, Humberto García Muñoz, Mª Ángeles Cabeza Rodríguez **(Hosp. 12 de Octubre). San Sebastián:** Irune Ruiz Díaz, José Ignacio Emparanza, Juan Pablo Sanz Jaka, Mª José Velásquez **(Hospital Donosita). Valencia:** Agustin LLopis González, María Morales **(Universidad de Valencia);** Carlos Camps, Cristina Caballero Díaz, Emilio Marqués Vidal, Francisco Sánchez Ballester, Joaquin Ulises Juan Escudero, Jorge Pastor Peidro, José López Torrecilla, Mª Macarena Ramos Campos, Miguel Martorell Cebollada **(Consorcio Hospital General U. de Valencia).**
